# Spatial distribution of sequential ventilation during mechanical ventilation of the uninjured lung: an argument for cyclical airway collapse and expansion

**DOI:** 10.1186/1471-2466-10-25

**Published:** 2010-05-05

**Authors:** Scott E Sinclair, Nayak L Polissar, William A Altemeier

**Affiliations:** 1 Department of Medicine, University of Tennessee Health Sciences Center, 956 Court Ave E222, Memphis, TN, 38163 USA; 2 The Mountain-Whisper-Light Statistical Consulting, 1827 23rd Ave East, Seattle, WA 98112 USA; 3 Department of Medicine, University of Washington, Box 358052, 815 Mercer St, Seattle, WA 98109 USA

## Abstract

**Background:**

Ventilator-induced lung injury (VILI) is a recognized complication of mechanical ventilation. Although the specific mechanism by which mechanical ventilation causes lung injury remains an active area of study, the application of positive end expiratory pressure (PEEP) reduces its severity. We have previously reported that VILI is spatially heterogeneous with the most severe injury in the dorsal-caudal lung. This regional injury heterogeneity was abolished by the application of PEEP = 8 cm H_2_O. We hypothesized that the spatial distribution of lung injury correlates with areas in which cyclical airway collapse and recruitment occurs.

**Methods:**

To test this hypothesis, rabbits were mechanically ventilated in the supine posture, and regional ventilation distribution was measured under four conditions: tidal volumes (V_T_) of 6 and 12 ml/kg with PEEP levels of 0 and 8 cm H_2_O.

**Results:**

We found that relative ventilation was sequentially redistributed towards dorsal-caudal lung with increasing tidal volume. This sequential ventilation redistribution was abolished with the addition of PEEP.

**Conclusions:**

These results suggest that cyclical airway collapse and recruitment is regionally heterogeneous and spatially correlated with areas most susceptible to VILI.

## Background

Lung injury and edema are well-documented consequences of mechanical ventilation with high distending pressures in multiple experimental models [1-3]. It has been observed that maintaining end-expiratory lung volume, at some level above functional residual capacity, with positive end-expiratory pressure (PEEP) can prevent/reduce this ventilator-induced lung injury (VILI). Multiple animal models employing high distending pressures and/or volumes have demonstrated marked reductions in lung injury when adequate PEEP is applied [1-5]. In a surfactant-depletion model, lung injury occurs in the absence of large tidal volumes and distending pressures if inadequate or absent PEEP is used [6]. However, applying PEEP above the lower inflection point of the inspiratory pressure-volume curve protected against injury [6]. The protective effect of PEEP has been attributed primarily to prevention of repeated airway collapse and expansion (RACE) [6,7], and to a lesser extent limitation of tidal excursion, and reduced cardiac output [8]. These observations, although not always directly translatable to the clinical management of humans with lung injury, shed light on how management of mechanical ventilatory support might potentially impact patient outcomes. It has recently been observed that outcomes can be improved in ARDS patients if distending pressures are limited by tidal volume reduction [9,10]. Although improved outcomes in ARDS patients ventilated with PEEP set above the lower inflection point of the inspiratory pressure-volume curve has been reported [10], a recent multi-center trial failed to show a survival advantage with a high-PEEP ventilation strategy [11]. Similar outcomes have been observed in two subsequent trials employing either oxygenation-based [12] or plateau pressure-based [13] PEEP protocols. However, another recent study documented significant heterogeneity in how patients with ALI/ARDS respond to higher PEEP levels [14]. They identified two populations of patients: those with a significant recruitable lung volume, and those with negligible recruitable volume. These intra-patient differences may suggest that some patients and not others may benefit from higher PEEP. Additionally, a recent study by Talmor and colleagues [15] examined the effect of higher PEEP based on a trans-pulmonary pressure-based protocol and found a strong trend toward improved survival in the higher PEEP group.

The RACE hypothesis has been called into question recently, favoring the tidal movement of fluid and/or foam in the airways as an explanation for the mechanical behavior of the injured lung during positive pressure ventilation [16]. Also, a recent model of saline lavage lung injury found that high tidal volume/low PEEP ventilation resulted in lung injury in the non-dependent lung regions in supine rats, suggesting atelectasis in the dependent lung zones shifts stretch-induced injury to the non-dependent lung and argues against repetitive collapse and expansion as a cause of VILI [17]. The current technological limitations of available imaging modalities preclude accurate real-time imaging of all but the most peripheral alveoli, therefore whether alveoli open and close during mechanical ventilation remains a point of contention.

We have recently shown in a rabbit model of VILI that lung injury is greater and more spatially variable with a dorso-caudal gradient in the absence of PEEP[18]. One potential mechanism for this finding is regional repeated airway collapse and expansion in dependent lung areas during tidal breathing promotes subsequent injury. We hypothesize that 1) cyclical airway closure and expansion or RACE occurs in the lungs of anesthetized rabbits mechanically ventilated in the supine posture without PEEP and 2) RACE would occur regionally in the dorso-caudal lung regions, spatially correlating with the previously reported regional distribution of VILI [18].

To test this hypothesis, we measured how relative ventilation regionally redistributes with increasing tidal volume in the lungs of mechanically ventilated rabbits by measuring the distribution of aerosolized fluorescent microspheres. This ventilation redistribution with increasing tidal expansion of the lungs is referred to as sequential ventilation and reflects dynamic regional changes in lung mechanics during inspiration. By measuring regional ventilation redistribution with increasing tidal volume under different PEEP conditions, we could infer whether dynamic regional changes in lung mechanics were related to changes in the lower part of the pressure-volume curve (i.e. cyclical airway closure at FRC followed by expansion and increased ventilation during inspiration) or to changes at the upper part of the pressure volume curve (i.e. alveolar overdistension and regionally reduced compliance resulting in decreased regional ventilation).

## Methods

### Animals Preparation

The University of Washington Animal Care Committee in accordance with National Institutes of Health guidelines approved all methods. New Zealand white rabbits (either sex, 2.4 to 2.8 kg) were sedated with intramuscular ketamine (30 mg/kg) and xylazine (7.5 mg/kg) to allow placement of a 20 ga catheter in each marginal ear vein. A surgical plane of anesthesia was then maintained with a continuous intravenous infusion of ketamine (0.05 mg/kg/hr) and xylazine (0.003 mg/kg/hr) for the remainder of the protocol. A 3.5 mm cuffless endotracheal tube was inserted via tracheotomy to allow positive pressure mechanical ventilation. Arterial catheters were placed in the left carotid (blood gas sampling and arterial pressure measurement) and right femoral (thermistor tipped catheter for thermodilution cardiac output) arteries. A catheter was inserted into the right internal jugular vein for pressure monitoring and administration of thermodilution injectate. Pancuronium bromide (0.15 to 0.2 mg/kg) was administered intravenously, after adequate anesthesia was established, to suppress spontaneous respiratory efforts. A 30-min stabilization period followed the completion of surgical preparation, after which baseline data were collected. During this period, animals were ventilated in pressure control mode (Servo 900 C; Semens-Elema, Stockholm, Sweden) with a 50% inspiratory time and no inspiratory pause. Tidal volume was set at 10 - 12 ml/kg, PEEP = 5 cm H_2_O and a respiratory rate to achieve P_a_CO2 = 35 - 45 mmHg.

### Physiologic Measurements

Data were recorded using Powerlab data acquisition software (AD-Instruments Castle Hill, New South Wales, Australia). Blood pressures (systemic arterial and right ventricular), heart rate, duplicate thermodilution cardiac output (Baxter Edwards SAT-2 Oximeter/Cardiac Output Computer, Irvine, CA), arterial blood gases (Radiometer ABL 5, Copenhagen, Denmark) and ventilatory parameters were measured for each experimental condition after a 20-minute stabilization period.

An in-line spirometer (KORR RSS 100; Medical Technologies Research Spirometry System, Salt Lake City, UT) was used to measure airway pressures and tidal volume. Plateau pressure was measured as the pressure achieved at the end of a 5-second end-inspiratory hold maneuver.

### Sequential Ventilation Measurement

On completion of surgery, stabilization, and collection of baseline data, each animal (n = 5) was subjected to 4 different experimental conditions combining high (12 ml/kg) or low (6 ml/kg) tidal volume with 8 cm H_2_0 or 0 cm H_2_O PEEP (Table 1). The rationale for choosing these four conditions is as follows. In the absence of PEEP, if dorso-caudal lung regions are closed at FRC and then subsequently recruited at some point during a tidal inspiration, relative ventilation to these regions will be greater with a larger tidal volume because the regions are open for a greater percentage of inspiration as compared with a smaller tidal volume. Thus, when measuring relative ventilation distribution at these two tidal volumes, there will be greater ventilation to the dorso-caudal regions and correspondingly less ventilation to ventro-cranial regions with the larger tidal volume. In contrast, if PEEP is set sufficiently high as to prevent cyclical airway collapse at FRC, relative regional ventilation distribution should be similar between a smaller tidal volume and a larger tidal volume.

**Table 1:** Physiological Response to Varying Mechanical Ventilation Strategies

Tidal Volume	6 ml/kg		12 ml/kg	
PEEP	0 cmH_2_O	8 cmH_2_O	0 cmH_2_O	8 cmH_2_O
Heart rate (min^-1^)	214 ± 45	219 ± 26	211 ± 25	230 ± 31
MAP (mmHg)	63 ± 18	47 ± 7	65 ± 9	52 ± 7
Mean RVP (mmHg)	14 ± 2	14 ± 1	13 ± 2	15 ± 1
Cardiac output (L·min^-1^)	0.34 ± 0.02	0.23 ± 0.05^†^	0.31 ± 0.05	0.25 ± 0.06
Peak P_AW _(cmH_2_O)	8 ± 1	16 ± 2^†^	13 ± 1*	30 ± 5*^†^
Plateau P_AW _(cmH_2_O)	7 ± 1	15 ± 2^†^	12 ± 2*	27 ± 2*^†^
Arterial pH	7.38 ± 0.10	7.38 ± 0.07	7.42 ± 0.04	7.43 ± 0.02
Arterial PO_2 _(torr)	69 ± 11	95 ± 6^†^	69 ± 12	94 ± 11^†^
Arterial PCO_2 _(torr)	42 ± 2	40 ± 5	41 ± 2	41 ± 1
A-aDO_2 _(torr)	19 ± 21	8 ± 9	25 ± 7	5 ± 8^†^

* p < 0.05 compared to other condition with same PEEP but different tidal volume; † p < 0.05 compared to other condition with different PEEP but same tidal volume. Mixed model used for statistical analysis with post-hoc comparisons using Tukey's HSD. Values presented are mean ± SD. MAP - mean arterial pressure, RVP - right ventricular pressure, P_AW _- airway pressure, A-aDO_2 _- alveolar-arterial oxygen difference.

The order of the four conditions was varied across experiments. Prior to each condition change, two static inflations to 40 cmH_2_0 were performed via the syringe technique and held for 20 seconds each to ensure a standard volume history between conditions. All animals were ventilated with room air throughout all experimental conditions and respiratory rate was adjusted at each condition to achieve P_a_CO_2 _of 35 to 45 mmHg.

Twenty minutes after each condition was established, physiologic data were recorded. Aerosolized 1-μm diameter fluorescent microspheres were administered over 5 min to measure regional ventilation for each condition as previously described [19]. A total of 4 different fluorescent colors (yellow, orange, orange-red, and red) were used. Each colored microsphere aerosol marked ventilation under one of the four conditions. By using four different aerosols, we can determine spatial ventilation distribution post-mortem for each of the four different ventilation conditions in each animal.

At the conclusion of each experiment, a sternotomy was performed, the main pulmonary artery and left atrium were cannulated, and the pulmonary vasculature was flushed with a dextran solution by gravity feed. The lungs were dissected from the chest cavity and dried inflated at 25-cmH_2_O. The dried lungs were fixed in rapid-setting foam, sliced, mapped, and diced into cubes of 1.5-2.0-cm^3^. Each piece was weighed, visually scored for airway and blood content, and soaked for 2 days in 1.5 ml of 2-ethoxyethyl acetate to extract the fluorescent dyes. The fluorescent signals for the four colors were measured in each piece with a fluorimeter (LS50B, Perkin-Elmer), corrected for background and spillover from adjacent signals [20], and converted to weight-normalized, relative ventilation signals as previously described [19].

### Data Analysis and Statistics

**Figure 1:**
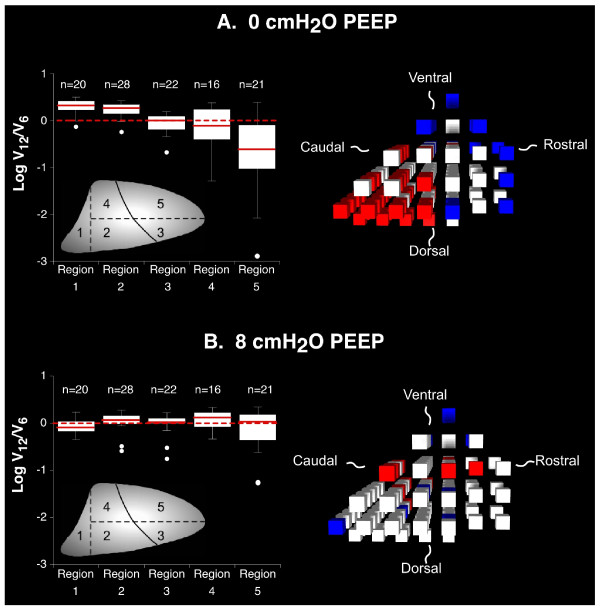
**Regional variability of sequential ventilation without and with positive end-expiratory pressure (PEEP)**. A) 0 cmH_2_O PEEP; B) 8 cmH_2_O PEEP. Figures on the left are box plots of the logarithm of the ratio of relative ventilation at 12 ml/kg tidal volume to relative ventilation at 6 ml/kg tidal volume (log V_12_/V_6_) by lung region for one representative animal. The box defines the inter-quartile range with a line indicating the median value. Whiskers indicate the plausible range of data with potential outliers identified as individual points. Figures on the right are 3-dimensional reconstructions of the lungs viewed from a lateral position. Red boxes indicate a 10% or greater increase and blue boxes represent a 10% or greater decrease in relative ventilation at 12 ml/kg compared with 6 ml/kg tidal volumes. White boxes represent regions with < 10% change in relative ventilation with tidal volume change. Inset: Lung Divisions: Each lung was divided into five regions. The first region included all lung pieces in all ventral-dorsal sections that were adjacent to the diaphragm at any point. The remaining lung was divided into four regions as indicated based on bisecting transverse and coronal planes.

All values are presented as means ± SD and all statistical analyses were done using JMP (SAS, Cary, NC). Comparisons of physiological parameters among the different ventilator conditions were made using a mixed model in which ventilator condition (the four combinations of tidal volume and PEEP) was the fixed effect and animal number was the random effect to account for correlations from repeated measures within each animal. For a given physiologic parameter there was one value for each animal for each of the four conditions. Post-hoc comparisons were made with Tukey's HSD. To assess for ventilation redistribution with change in tidal volume, the base 10 logarithm of the ratio of the relative ventilation with 12 ml/kg tidal volume to ventilation with 6 ml/kg was calculated for each lung piece. For a given piece of lung, if the log ratio was greater than 0, the relative ventilation to that lung piece increased as tidal volume increased; conversely if the log ratio was less than 0, the relative ventilation to that lung piece decreased as tidal volume increased. To evaluate for regional changes in relative ventilation distribution, fluorescent measurements were clustered into five regions based on dome of the diaphragm, major fissure, and a mid-sagittal division as illustrated in Figure 1. This clustering correlates with our previously published data on regional lung injury [18]. Because measurements for all four ventilatory conditions were made in each animal and because the log ratio of ventilation with a tidal volume of 12 ml/kg to a tidal volume of 6 ml/kg is not independent among the five regions within any given animal, we could not perform a standard statistical analysis looking at differences between the five regions at different PEEP levels. To quantify the effect of PEEP on ventilation distribution, we calculated the median of the log ratio of ventilation for each region (Figure 1) and then the standard deviation of these medians across the five different regions at both PEEP levels. A lower standard deviation represents little difference in median ventilation distribution across the five regions between tidal volumes; whereas, a higher standard deviation represents greater heterogeneity of relative ventilation distribution across the five regions between tidal volumes. A paired t-test was used to compare the log_10_-transformed standard deviation of median log ratios between PEEP levels.

## Results

### Measurement of sequential ventilation distribution

Five animals were studied using the sequential ventilation experimental protocol. Several of the hemodynamic parameters were similar among the four ventilation conditions, although both mean arterial pressure (MAP) and cardiac output were lower during ventilation with PEEP for both tidal volumes (p = 0.08 and 0.01 for PEEP effect on MAP and cardiac output, respectively, at 6 ml/kg). Airway pressures were higher both with larger tidal volumes and with the addition of PEEP (Table 1), including statistically significant differences for three out of four comparisons. Arterial oxygenation was statistically higher during ventilation with PEEP (Table 1) for both tidal volumes; there were no statistically significant differences in arterial pH or PCO_2 _among the four conditions.

**Figure 2:**
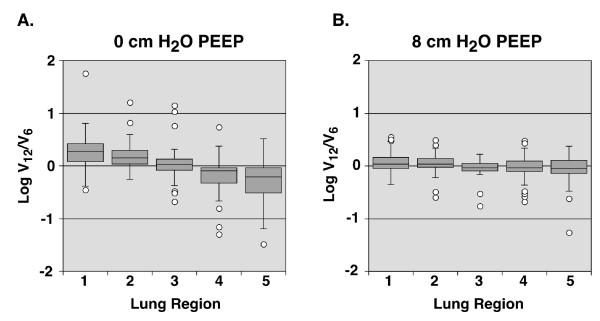
**Composite log ratios of regional ventilation**. Box plots of composite log ratio ventilation (log V_12_/V_6_) data for all pieces from all five animals, divided by lung region. A) 0 cmH_2_O PEEP; B) 8 cmH_2_O.

An average of 73 ± 21 lung pieces were obtained from each animal for measurement of regional ventilation across the four conditions. In the absence of PEEP, four out of five animals demonstrated a pattern in which relative ventilation increased to dorsal-caudal lung regions (regions 1 and 2) and decreased to ventral-rostral regions (regions 4 and 5) when tidal volumes were increased from 6 ml/kg to 12 ml/kg (Figure 1A). When ventilation distribution was measured in the presence of 8 cmH_2_O PEEP, there was no clear difference in relative ventilation distribution between 6 ml/kg and 12 ml/kg tidal volume ventilation in any animals (Figure 1B). A composite graph of all data points across the five animals demonstrates the consistency of this finding (Figure 2). The addition of PEEP resulted in a strong trend towards a reduced standard deviation of median log ratios of ventilation with a tidal volume of 12 ml/kg to a tidal volume of 6 ml/kg across the five regions (0.114 ± 0.105 vs. 0.238 ± 0.150, p = 0.08).

**Figure 3:**
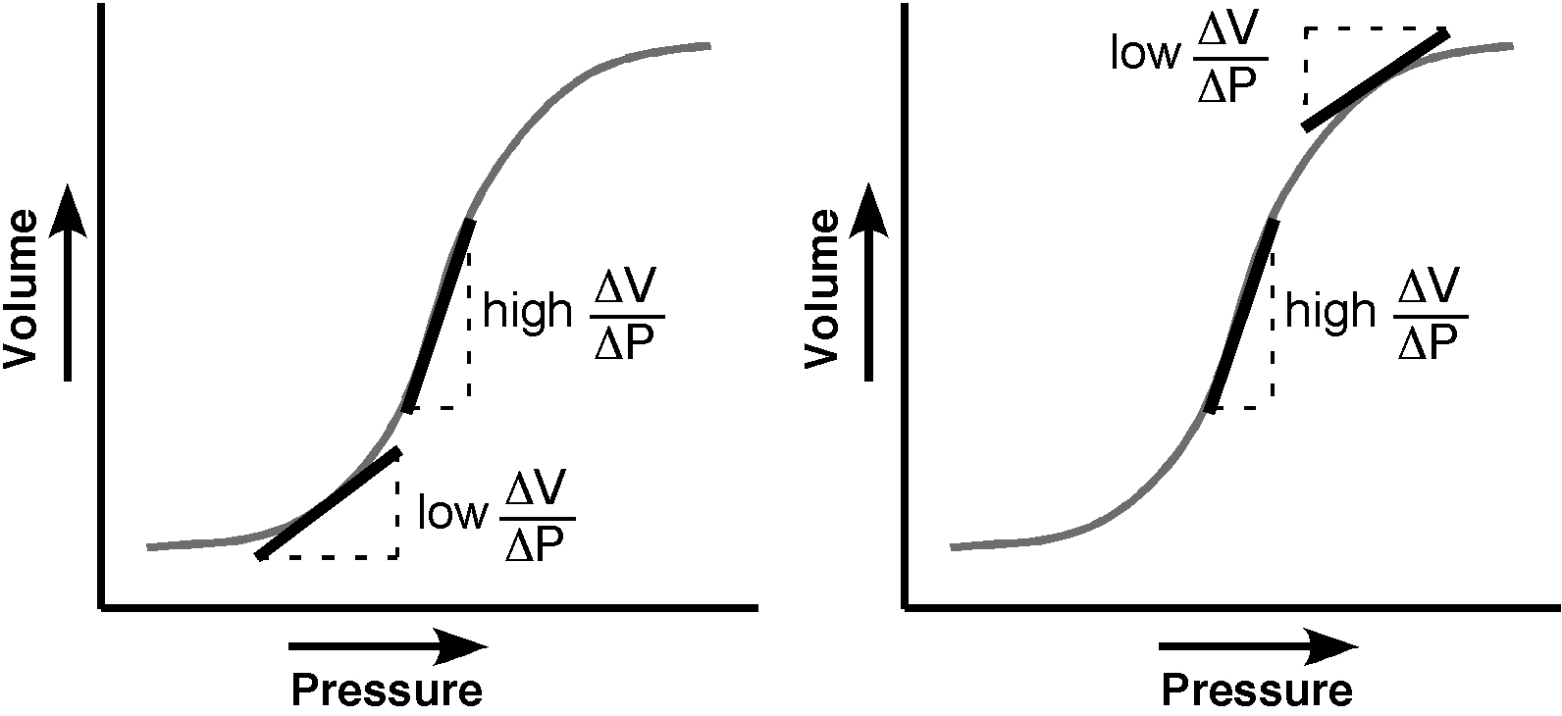
**Theoretical pressure-volume curves illustrating changing compliance with increasing tidal volume**. Compliance may either increase as collapsed alveoli are recruited (A) or decrease as alveoli become overdistended (B).

In summary, animals ventilated in the supine posture without PEEP demonstrated sequential redistribution of ventilation towards dorsal-caudal lung regions as tidal volume increased suggesting increasing regional compliance in dorsal-caudal lung regions relative to ventral-cranial regions. This sequential redistribution of ventilation was attenuated with the application of PEEP. This finding further suggests that the mechanism for the change in relative regional compliance was recruitment and increased compliance in dorsal-caudal lung, rather than over-distension and decreased compliance in ventral-cranial lung.

## Discussion

In this study, we hypothesized that, in anesthetized, mechanically ventilated, supine rabbits, there are regional areas of lung, which undergo cyclical airway collapse at FRC and expansion during tidal inspiration (RACE) in the absence of PEEP and that this results in spatial redistribution of relative ventilation with increasing tidal volume. We further hypothesized that RACE would spatially correlate with the most regionally severe lung injury observed in a prior study of VILI. We found that relative ventilation increased to dorso-caudal lung regions as tidal volume increased and that this regional ventilation redistribution with increasing tidal volume was ameliorated with the addition of positive end-expiratory pressure. These data support regional cyclical airway closure at FRC and recruitment with tidal inspiration in the absence of PEEP, based on the following assumptions:

1. A regional increase in relative ventilation with an increase in tidal volume implies that local compliance is increasing relative to other lung regions.

2. Local lung compliance is roughly constant between volumes associated with either atelectasis (lower inflection point on a pressure-volume curve) or full inflation (upper inflection point on a pressure-volume curve - Figure 3).

3. Regional lung compliance can increase relative to other lung regions either because of local recruitment and improved compliance (Figure 3A) or because of over distension and reduced compliance in remote lung regions (Figure 3B).

4. If recruitment of dependent alveoli, which collapse at FRC, is responsible for redistribution of ventilation to dorso-caudal lung with increasing tidal volume, then application of PEEP should reduce this redistribution. However, if over distension of non-dependent alveoli is responsible for ventilation redistribution to dorso-caudal lung, then application of PEEP should augment this redistribution.

Although we have not directly visualized RACE, which is not obviously feasible, our data support a spatial distribution of cyclical airway collapse and recruitment, which correlates with our previously reported spatial distribution of lung injury [18]. Because RACE is implicated as one cause of ventilator-induced lung injury [6,18,21-23], these findings further support regional RACE in uninjured, supine rabbits undergoing mechanical ventilation. All non-invasive measurements of volume change or alveolar density are indirect estimates of regional ventilation subject to erroneous interpretation. Using dynamic measurement of regional ventilation by the novel method of labeled aerosol deposition, we have demonstrated for the first time that regional ventilation redistributes with increasing tidal volume, suggesting dynamic regional changes in lung mechanics. Furthermore, by measuring sequential ventilation at different PEEP levels, we have shown that RACE is the most likely explanation for these dynamic changes in regional lung mechanics.

We have previously reported a dramatic dorsal-ventral and cranial-caudal gradient of lung injury severity in a rabbit model of VILI with supine ventilation in the absence of PEEP. The region with the most severe injury was the dorsal-caudal lung. With the application of 8 cmH_2_O of PEEP however, no such gradient is observed, rather a less severe, more homogeneously distributed injury pattern occurs, despite identical end inspiratory pressures [18]. The application of PEEP has been shown to ameliorate VILI in a number of experimental models [1,3,4,6,24-26]. One potential explanation for the protective effects of PEEP is the prevention of tidal collapse and re-expansion of distal lung units. In the supine posture, lower alveolar volumes at FRC may predispose dependent lung regions to cyclical collapse. Muscedere et. al. [6] demonstrated epithelial injury in distal airways and alveoli, even with low tidal volumes (6 ml/kg), when isolated, unperfused rat lungs were allowed to deflate to volumes below the lower inflection point of the pressure-volume curve during exhalation. Although compelling, these findings must be interpreted in the context of an *ex vivo*, unperfused model which will favor tidal airway collapse and minimize edema formation in comparison with an intact animal model [16].

Distal airways and alveoli are not generally felt to be at risk of tidal collapse and re-expansion *in vivo *in uninjured lungs. In the current study, we examined this during mechanical ventilation in the supine posture by demonstrating sequential distribution of ventilation to the dorsal-caudal lung regions with increasing tidal volume, which was abolished by the application of PEEP. In the supine posture, a reduction was seen in relative ventilation to the dorsal-caudal region. One potential explanation for this is that the FRC is below the closing volume in this region at end-exhalation, resulting in cyclical airway closure. This would result in sequential ventilation with no inspired gas going to this lung region during the initial phase of tidal breathing, followed by recruitment and ventilation in the latter phase of each breath. To test this, we subjected uninjured animals to small (6 ml/kg) and large (12 ml/kg) tidal volume ventilation and measured relative regional ventilation distribution with inhaled fluorescent microspheres. We found a relative increase in ventilation to the dorsal-caudal lung and decreased ventilation to the ventral-cranial lung with larger tidal volumes. Two likely explanations for this observation are 1) alveoli in the dorsal-caudal lung were recruited with increased tidal volume or 2) ventral-cranial regions were over-distended with increased tidal volume, became less compliant, and ventilation was re-distributed to the dorsal-caudal lung. The effect of PEEP on the distribution of ventilation favors the former explanation. The application of PEEP = 8 cm H_2_O eliminated the observed sequential ventilation in the dorsal-caudal lung, consistent with recruitment of alveoli in this region which allowed the same relative ventilation to be delivered independent of tidal volume.

There are several limitations to the current study. The tidal volume used to induce VILI in our previous study [18] as well as other studies of VILI were much greater than the tidal volumes used in the measurement of ventilation distribution. The lower tidal volumes were chosen to avoid inducing lung injury during the experiment because aerosol deposition is unlikely to reflect ventilation distribution in the presence of spatially heterogeneous pulmonary edema secondary to differences in particle deposition. We did not measure ventilation distributions in the prone posture to identify whether or not sequential ventilation distribution was diminished. However, we have previously shown in a porcine model that posture change from supine to prone increases dorsal-caudal ventilation consistent with reduced airway collapse in these regions [19]. Additionally, reduced sequential ventilation and airway closure in the prone posture compared with supine posture has been previously shown in dogs with normal lungs by single breath washout test [27]. We have also ventilated animals in the prone posture in an identical fashion to that previously reported in supine animals. With supine ventilation, the most severe injury occurred in the dorsal-caudal lung while less severe injury was seen in the cranial-ventral region. In contrast, prone ventilation produced a more modest and homogeneously distributed injury pattern [4]. These results are similar to that of previous observations in both oleic acid injured [22] and normal dogs [21]. One potential explanation for the protective effects of prone posture is the prevention of tidal collapse and re-expansion of distal lung units. In the supine posture, lower alveolar volumes at FRC may predispose dependent lung regions to cyclical collapse. The more uniform pleural pressure gradient along the dorsal-ventral axis, in the prone posture, results in higher regional FRC in dependent lung zones [28] which may limit tidal collapse and recruitment of airways, and thus reduce VILI.

## Conclusions

We previously reported in a rabbit model of VILI that lung injury is greater and more spatially heterogeneous in the supine posture as compared with the prone posture. One potential mechanism for this finding is regional repeated airway collapse and expansion during tidal breathing. To evaluate whether or not this occurred in the absence of lung injury with supine positioning, we measured regional ventilation at different tidal volumes in the presence and absence of PEEP. We found evidence of sequential ventilation distribution towards dorsal-caudal lung and away from ventral-cranial lung with increasing tidal volume. This sequential ventilation pattern was attenuated with the addition of PEEP compatible with RACE. We speculate that RACE occurs in normal lung with supine ventilation and in the absence of PEEP and that this may contribute to the development of lung injury.

## List of Abbreviations

PEEP: Positive end expiratory pressure; VILI: Ventilator-induced lung injury; RACE: Repeated airways collapse and expansion; P_a_CO_2_: Partial pressure of arterial carbon dioxide; P_a_O_2_: Partial pressure of arterial oxygen

## Competing interests

The authors declare that they have no competing interests.

## Authors' contributions

SS and WA designed and performed the experiments, analyzed the data and wrote the manuscript. NP designed the statistical methods and reviewed and edited the manuscript. All authors read and approved the final manuscript.

## Pre-publication history

The pre-publication history for this paper can be accessed here:

http://www.biomedcentral.com/1471-2466/10/25/prepub

## References

[B1] Dreyfuss D Basset G Soler P Saumon G Intermittent positive-pressure hyperventilation with high inflation pressures produces pulmonary microvascular injury in rats Am Rev Respir Dis 1985 132 4 880 884 3901844 10.1164/arrd.1985.132.4.880

[B2] Dreyfuss D Soler P Basset G Saumon G High inflation pressure pulmonary edema. Respective effects of high airway pressure, high tidal volume, and positive end-expiratory pressure Am Rev Respir Dis 1988 137 5 1159 1164 3057957 10.1164/ajrccm/137.5.1159

[B3] Webb HH Tierney DF Experimental pulmonary edema due to intermittent positive pressure ventilation with high inflation pressures. Protection by positive end-expiratory pressure Am Rev Respir Dis 1974 110 5 556 565 4611290 10.1164/arrd.1974.110.5.556

[B4] Sinclair S Souders J Hlastala M Severity and distribution of ventilator-induced lung injury is altered by PEEP, prone position, and respiratory frequency in normal rabbits Am J Respir Crit Care Med 1998 157 3 A107

[B5] Sinclair SE Kregenow DA Lamm WJ Starr IR Chi EY Hlastala MP Hypercapnic acidosis is protective in an in vivo model of ventilator-induced lung injury Am J Respir Crit Care Med 2002 166 3 403 408 10.1164/rccm.200112-117OC 12153979

[B6] Muscedere JG Mullen JB Gan K Slutsky AS Tidal ventilation at low airway pressures can augment lung injury Am J Respir Crit Care Med 1994 149 5 1327 1334 8173774 10.1164/ajrccm.149.5.8173774

[B7] Russell J Slutsky A Lemaire F Ramsay G Mancebo J Richard C International Concensus Conference in Intensive Care Medicine: Ventilator-associated Lung Injury in ARDS Am J Resp Crit Care Med 1999 160 2118 2124 10588637 10.1164/ajrccm.160.6.ats16060

[B8] Luce JM The cardiovascular effects of mechanical ventilation and positive end-expiratory pressure Jama 1984 252 6 807 811 10.1001/jama.252.6.807 6379209

[B9] Ventilation with lower tidal volumes as compared with traditional tidal volumes for acute lung injury and the acute respiratory distress syndrome. The Acute Respiratory Distress Syndrome Network N Engl J Med 2000 342 18 1301 1308 10.1056/NEJM200005043421801 10793162

[B10] Amato MB Barbas CS Medeiros DM Magaldi RB Schettino GP Lorenzi-Filho G Kairalla RA Deheinzelin D Munoz C Oliveira R Effect of a protective-ventilation strategy on mortality in the acute respiratory distress syndrome N Engl J Med 1998 338 6 347 354 10.1056/NEJM199802053380602 9449727

[B11] Brower RG Lanken PN MacIntyre N Matthay MA Morris A Ancukiewicz M Schoenfeld D Thompson BT Higher versus lower positive end-expiratory pressures in patients with the acute respiratory distress syndrome N Engl J Med 2004 351 4 327 336 10.1056/NEJMoa032193 15269312

[B12] Mercat A Richard JC Vielle B Jaber S Osman D Diehl JL Lefrant JY Prat G Richecoeur J Nieszkowska A Positive end-expiratory pressure setting in adults with acute lung injury and acute respiratory distress syndrome: a randomized controlled trial Jama 2008 299 6 646 655 10.1001/jama.299.6.646 18270353

[B13] Meade MO Cook DJ Guyatt GH Slutsky AS Arabi YM Cooper DJ Davies AR Hand LE Zhou Q Thabane L Ventilation strategy using low tidal volumes, recruitment maneuvers, and high positive end-expiratory pressure for acute lung injury and acute respiratory distress syndrome: a randomized controlled trial Jama 2008 299 6 637 645 10.1001/jama.299.6.637 18270352

[B14] Grasso S Fanelli V Cafarelli A Anaclerio R Amabile M Ancona G Fiore T Effects of high versus low positive end-expiratory pressures in acute respiratory distress syndrome Am J Respir Crit Care Med 2005 171 9 1002 1008 10.1164/rccm.200407-940OC 15665322

[B15] Talmor D Sarge T Malhotra A O'Donnell CR Ritz R Lisbon A Novack V Loring SH Mechanical ventilation guided by esophageal pressure in acute lung injury N Engl J Med 2008 359 20 2095 2104 10.1056/NEJMoa0708638 19001507 PMC3969885

[B16] Hubmayr RD Perspective on lung injury and recruitment: a skeptical look at the opening and collapse story Am J Respir Crit Care Med 2002 165 12 1647 1653 10.1164/rccm.2001080-01CP 12070067

[B17] Tsuchida S Engelberts D Peltekova V Hopkins N Frndova H Babyn P McKerlie C Post M McLoughlin P Kavanagh BP Atelectasis causes alveolar injury in nonatelectatic lung regions Am J Respir Crit Care Med 2006 174 3 279 289 10.1164/rccm.200506-1006OC 16675780

[B18] Sinclair SE Chi E Lin HI Altemeier WA Positive end-expiratory pressure alters the severity and spatial heterogeneity of ventilator-induced lung injury: an argument for cyclical airway collapse Journal of critical care 2009 24 2 206 211 10.1016/j.jcrc.2008.04.005 19327294 PMC2720092

[B19] Altemeier WA McKinney S Krueger M Glenny RW Effect of posture on regional gas exchange in pigs J Appl Physiol 2004 97 6 2104 2111 10.1152/japplphysiol.00072.2004 15298981

[B20] Schimmel C Frazer D Glenny RW Extending fluorescent microsphere methods for regional organ blood flow to 13 simultaneous colors Am J Physiol Heart Circ Physiol 2001 280 6 H2496 2506 11356604 10.1152/ajpheart.2001.280.6.H2496

[B21] Broccard A Shapiro RS Schmitz LL Adams AB Nahum A Marini JJ Prone positioning attenuates and redistributes ventilator-induced lung injury in dogs Crit Care Med 2000 28 2 295 303 10.1097/00003246-200002000-00001 10708156

[B22] Broccard AF Shapiro RS Schmitz LL Ravenscraft SA Marini JJ Influence of prone position on the extent and distribution of lung injury in a high tidal volume oleic acid model of acute respiratory distress syndrome Crit Care Med 1997 25 1 16 27 10.1097/00003246-199701000-00007 8989171

[B23] D'Angelo E Pecchiari M Baraggia P Saetta M Balestro E Milic-Emili J Low-volume ventilation causes peripheral airway injury and increased airway resistance in normal rabbits J Appl Physiol 2002 92 3 949 956 11842025 10.1152/japplphysiol.00776.2001

[B24] Argiras EP Blakeley CR Dunnill MS Otremski S Sykes MK High PEEP decreases hyaline membrane formation in surfactant deficient lungs Br J Anaesth 1987 59 10 1278 1285 10.1093/bja/59.10.1278 3314957

[B25] Corbridge TC Wood LD Crawford GP Chudoba MJ Yanos J Sznajder JI Adverse effects of large tidal volume and low PEEP in canine acid aspiration Am Rev Respir Dis 1990 142 2 311 315 2200314 10.1164/ajrccm/142.2.311

[B26] Tremblay L Valenza F Ribeiro SP Li J Slutsky AS Injurious ventilatory strategies increase cytokines and c-fos m-RNA expression in an isolated rat lung model J Clin Invest 1997 99 5 944 952 10.1172/JCI119259 9062352 PMC507902

[B27] Tomioka S Kubo S Guy HJ Prisk GK Gravitational independence of single-breath washout tests in recumbent dogs J Appl Physiol 1988 64 2 642 648 10.1063/1.341954 3372422

[B28] Kaneko K Milic-Emili J Dolovich MB Dawson A Bates DV Regional distribution of ventilation and perfusion as a function of body position J Appl Physiol 1966 21 3 767 777 5912746 10.1152/jappl.1966.21.3.767

